# Dietary zinc intake and its determinants among Ethiopian children 6–35 months of age

**DOI:** 10.1186/s40795-018-0237-8

**Published:** 2018-08-09

**Authors:** Girmay Ayana, Tibebu Moges, Aregash Samuel, Tsehai Asefa, Solomon Eshetu, Aweke Kebede

**Affiliations:** Ethiopian Public Health Institute, Addis Ababa, Ethiopia

**Keywords:** Stunting, Dietary intake, Zinc, Ethiopia

## Abstract

**Background:**

Adequate zinc intake is essential for the growth and neurobehavioral development of young children. Zinc deficiency in children is recognized as risk factor for stunting. In Ethiopia, 38% of children under five years of age are stunted. This analysis was conducted to measure dietary zinc intake and to identify its determinants among children 6–35 months of age to design appropriate intervention.

**Methods:**

Nationally and regionally representative data available from 6752 children 6–35 months of age from the Ethiopian national food consumption survey were analyzed. A multivariate model was used to identify determinants of dietary zinc intake.

**Results:**

*W*e found low dietary zinc intake among children 6–35 month age. National average dietary zinc intake was 1.74 mg/day. Socio-economic status, maternal education, and maternal age were positively associated with dietary zinc intake, while the number of children under 5 years-of-age in a household was negatively associated with dietary zinc intake (*p* < 0.0001). Children reportedly sick in the previous 2 weeks were most likely to have low dietary zinc intake (*p* < 0.0001).

**Conclusion:**

The observed low dietary zinc intake in Ethiopian children has a significant association with health status of children, providing evidence for nutrition and health planners to emphasize on promoting consumption of zinc rich foods and preventing morbidity from common infections.

## Background

Adequate zinc intake is essential for the growth and neurobehavioral development of young children. Low dietary zinc intake is the main cause of zinc deficiency [1]. When zinc rich source foods are not available on a routine diet, zinc deficiency (insufficient zinc to meet the needs of the body) develops over time and persists until changes are made in the diet [2, 3]. Recent estimates show that17.3% of the world’s and 23.9% of Africa’s population is at risk of zinc deficiency [4] and 26% Sub-Saharan Africa people have inadequate access of zinc [5]. Zinc uptake depends on the amount of zinc consumed and the presence of dietary phytate [6]. Even though varied food sources contain zinc, the highest concentrations found in animal-source foods [5]. In developing countries due to economic, cultural and religious constraints, animal source foods consumption is limited and often inaccessible to lower-income households [7].

The first 2 years of life is a crucial period for children growth [4]. Nutrient-rich and diverse diets are essential for children to meet their nutrient needs and support optimal growth [8]. Stunting (height for age < − 2 SD) is a well-established child health indicator for chronic nutritional problems related to environmental and socio-economic circumstances [6, 9]. In the absence of direct measure, dietary zinc intake and stunting prevalence are good indicators of a population’s risk of zinc deficiency [10]. Because zinc is required for normal linear growth, an elevated prevalence of stunting can be considered as suggestive evidence of an increased risk of zinc deficiency in a population [11].

The high intakes of phytate relative to zinc in the diet of developing countries children, indicate that these children are at great risk for inadequate zinc intake [12].The diets of people living in developing countries are often low in animal products and high in plants or cereal meals high in inhibitors [13]. As a result, the amount of zinc available for absorption from such diets is low, and probably can be the primary cause of zinc deficiency [7].

In Ethiopia animal source food consumption is very low and low quality diet consumption is a common practice. Dietary zinc intake and determining factors among Ethiopia children at national level has not been assessed and reported.Our study aimed at measuring dietary zinc intake and identifying its determinants among children 6–35 months of age in Ethiopia.

## Methods

Ethiopia has nine regions and two city administrations. The Ethiopian food consumption survey (ENFCS) was a nationally representative population based individual level survey conducted in 2013 to provide evidence about food consumption in Ethiopia. Data was collected on 6752 children from 8267 households.The target population of interest was young children (6–35 months of age) and data was collected on socio-demographic information, anthropometric measurements and dietary intake(using 24-h dietary recall). The 24 h dietary recall was primarily administered to mother-child pair living in the selected household. For children living without mothers, adult female caregiver was involved. Thus, data were collected from a mother or adult female caregiver and one child in the selected age ranges in each household. For estimation of nutrient intakes, Ethiopian food composition tables were used as a basis for food composition database. To fill missing data gaps in the food composition tables, supplementary datasets and published values were used. Measurements of weight, and length/height were used to assess the nutritional status of children 6-35 months. For analysis purpose, maternal education and maternal age has been categorized. Socioeconomic status of households was also categorized in quartiles. We used bivariate and multivariate analysis model to determine the association of our independent variables with dietary zinc intake,.

In order to ensure informed voluntary participation, the proposal has been submitted for approval to Scientific and Ethical Review Office (SERO) of Ethiopian Public Health Institute and got the final approval. The aim of the survey, as well as the type of measurements to be taken were explained prior to commencing measurements. Verbal consent was obtained from the adult participants, with permission for participation of children. All participants were informed of their option to withdraw from participation at any time.

Regular supervision was undertaken at various levels to ensure the quality of the data. To confirm the quality of data, supervisors had conducted truncated spot-checks in 10% of interviewed households.

In this paper we report findings on determinants of dietary zinc intake among children 6–35 months of age as well as the association between dietary zinc intake and height for age. We used data available from children 6–35 months of age for analysis of dietary zinc intake. A general linear model was used to identify determinants of dietary zinc intake and to determine association between height for age and dietary zinc intake in children aged 6–35 months in Ethiopia. Zinc intake was not normally distributed therefore, during analysis we transformed the data to the log10 form. This was back transformed in the result writing.

## Results

### Socio demographic characteristics of household with children 6–35 month months age

Majority of mothers/care givers had low formal education level (58.7%). Women were more likely to be involved in unpaid family work than head of households. The respondents were fairly evenly distributed across all wealth indexes (Table 1).

**Table 1:** Socioeconomic and demographic characteristics of households with children 6–35 month age, Ethiopia

Socioeconomic and demographic characteristics	n	Percent
Household head level of education	4954	
No education		34.4
Can read and write		8.6
Primary, 1st cycle		11.8
Primary, 2nd cycle		20.9
High school		17.3
Tertiary level		6.9
Head of household employment	4904	
Self employed		77.1
Employed		8.8
Public service worker		4.7
Unpaid family worker		1.3
Other		8.1
Women’s relationship to child	7640	
Biological Mother		95.0
Caregiver		5.0
Mother’s/women caregiver’s employment	7636	
Self employed		43.8
Employed		2.8
Public service worker		1.4
Unpaid family worker		43.1
Other		9.0
Mother’s/women’s caregiver’s level of education	7835	
No education		58.7
Can read and write		2.8
Primary, 1st cycle		10.6
Primary, 2nd cycle		12.2
High School		11.4
Other		4.3
Wealth index	7906	
Poorest		23.1
Second		20.4
Middle		19.2
Fourth		18.0
Richest		19.3

### Children nutritional status

High stunting prevalence was found in the agrarian regions and relatively low stunting prevalence was found in the pastoralist community. The prevalence of stunting was above the national level of 38.7% in the South Nations Nationalities and People (40.7%), Amhara (44.9%) and Tigray (47.7%) regions. Lowest stunting prevalence was found in Gambela (24.4%) and Addis Ababa (19.4%) regions (Table 2).

**Table 2:** Prevalence of under nutrition in children 6–35 month age in Ethiopia

Region	Stunting Prevalence (%)			Wasting Prevalence (%)			Underweight Prevalence (%)		
	N	<-3SD	<-2SD	N	<-3SD	<-2SD	n	<-3SD	<-2SD
Tigray	640	18.1	47.7	639	1.7	8.9	638	6.8	28.4
Afar	497	17.7	36.1	492	6.6	26.0	494	16	38.6
Amhara	825	21	44.9	822	1.4	8.1	823	7.6	27.9
Oromiya	921	15.3	34.9	918	1.6	8.9	920	8	22.4
Somali	615	11.2	27.4	612	3.2	13.1	614	7.2	22.5
B/Gumuz	501	18.8	38.4	500	3.9	15.1	501	11.2	27.6
SNNP	893	18.6	40.7	891	1.2	6.3	893	7	21.9
Gambella	415	10.1	24.4	415	3.1	13.9	415	6.3	21.5
Harari	383	12.9	31.0	382	1.2	8.2	383	4.9	20.8
A/ Ababa	572	6.6	19.4	570	0.7	3.2	572	2	7.6
Dire Dawa	440	10.8	27.7	440	2.6	10.6	440	5	19.9
Total	6702	17.2	38.7	6681	1.6	8.4	6693	7.5	23.8

### Children average dietary zinc intake (mg/24 h) in Ethiopia

From the bivariate analysis, We found no difference between urban and rural children in mean dietary zinc intake(1.51 versus 1.65, *P* = 0.126). Dietary zinc intake varied with socioeconomic status of the households. Maternal education has association with zinc intake. Non breast feeding children had a higher dietary zinc intake than breastfeeding(Table 3).

**Table 3:** Average dietary zinc intake (mg/24 h) among children 6–35 months of age, Ethiopia

Variables		N	Average Zn intake (mg/24 h)	SE(95% CI)	*p*-value
Residence	Urban	1757	1.51	1.00 [1.5, 1.51]	0.126
	Rural	4831	1.65	1.00 [1.64, 1.65]	
SES	Poorest	1315	1.48	1.00 [1.48, 1.48]	< 0.0001
	Second	1342	1.61	1.00 [1.61, 1.61]	
	Middle	1319	1.48	1.00 [1.48, 1.49]	
	Fourth	1343	1.64	1.00 [1.63, 1.64]	
	Richest	1269	1.68	1.00 [1.68, 1.69]	
Mother’s education	Illiterate	4001	1.00	1.00 [1.00, 1.00]	< 0.0001
	Can read and write	168	1.00	1.00 [1.00, 1.00]	
	Primary school, (G:1–4)	698	1.56	1.00 [1.55, 1.56]	
	Primary school (G:5–8)	901	1.47	1.00 [1.46, 1.47]	
	High school	643	1.61	1.00 [1.61, 1.61]	
	other	177	1.58	1.00 [1.57, 1.58]	
Child Breast feeding	No	2167	1.71	1.00 [1.71, 1.71]	< 0.0001
	Yes	4421	1.00	1.00 [1.00, 1.00]	
Child sex	Male	3525	1.88	1.00 [1.88, 1.89]	0.141
	Female	3063	1.32	1.00 [1.32,1.32]	

### Mean dietary zinc intake among children aged 6–35 months (*N* = 6752)

**Fig. 1:**
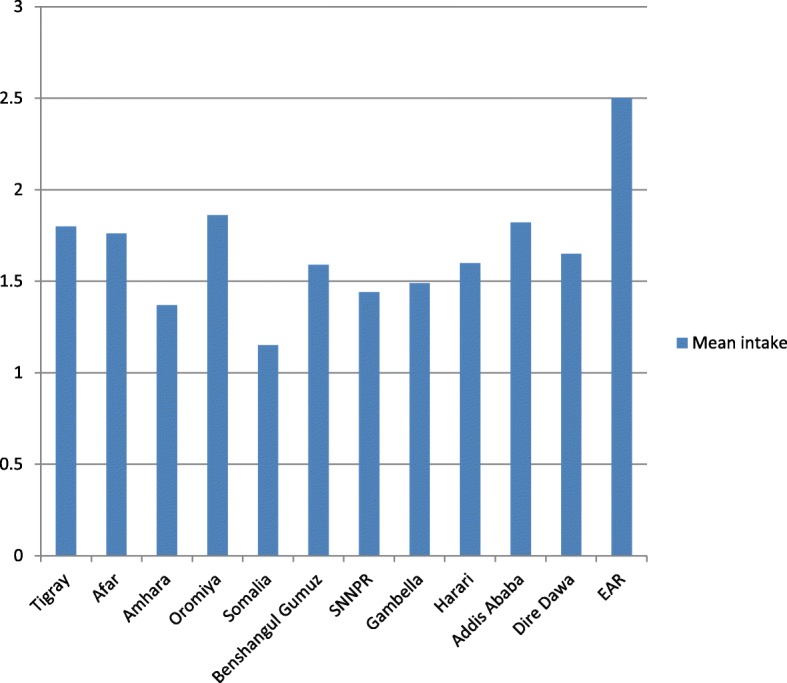
Mean dietary zinc intake of children 6–35 months of age by regions, Ethiopia

A total of 6752 data were analyzed to measure dietary zinc intake among children 6–35 months of age in all regions/city administrations. We found low dietary zinc consumption among children over the country. Nationally, mean dietary zinc intake of children 6–35 months of age was 1.74 mg/day. Children’s mean dietary zinc intake was highest in Oromia region and Addis Ababa city. The lowest mean dietary zinc intake found in Somali and Amhara region (Fig. 1).

### Determinants of dietary zinc intake in children 6–35 months age

Maternal age, female relationship to a child and child sickness in the previous two weeks had a significant association with the children dietary zinc intake (*p* value< 0.0001). The intake of dietary zinc has no association with household socioeconomic status and child sex. Place of residence/region has also a significant association with dietary intake (Table 4).

**Table 4:** Determinants of mean dietary zinc intake among children 6–35 month in Ethiopia

Independent variable	Regression coefficient	*p*-value
Child age	−0.02	< 0.0001
Number of children < 5 yrs	−0.11	0.71
Female relationship to child	0.14	< 0.0001
Child sex	0.01	0.016
Child sickness in previous 2 weeks	0.13	< 0.0001
Place of residence (urban/rural)	0.04	0.004
SES quintile	0.06	0.19
Mother’s age	0.02	< 0.0001
Mothers education status	0.41	0.004
Head of household education status	0.35	0.84
Region	0.16	< 0.0001

## Discussion

We found low mean dietary zinc intake in Ethiopian children. Children from illiterate mothers had lower dietary zinc intake than those from educated mothers and current breastfeeding children had lower dietary zinc intake than non breastfed children. Children’s place of residence has no significant difference on their dietary zinc intake. The main determinants of dietary intake in Ethiopian children were child health status, child age, maternal education status and child relationship to a mother.

Children nutritional status in Ethiopia is generally poor, and under-nourishment is one of the major health problems. The prevalence of stunting and underweight is considerably high in Ethiopia [14]. Children from developing countries has limited access to animal source food [15] and consume less amount of zinc which resulted in children’s restricted growth [16]. In Ethiopia cereals consumption on average accounts for more than 60% of the total households [17] and low dietary diversity and inappropriate child feeding practices [18] had contribution to the unacceptable stunting prevalence. Increased consumption of animal-source foods and phytate reduction are the preferred approaches to enhance content and bioavailability of Zn in the diets of rural households in developing countries [16]. Consumption of animal source foods and increasing dietary diversification could reduce stunting in children [19].Children from lower-income settings with inadequate intake and poor absorption from cereal-based complementary foods had a higher risk of zinc deficiency [20]. A study conducted in southern Ethiopia had confirmed that zinc intakes in pregnant women was very low and it was below reported level in many developing countries [21]. Our study also revealed inadequate dietary zinc intake in Ethiopian children. Similar to our finding a study conducted in Uganda has also shown that zinc intake does not meet the requirement in children [22].

Our study indicated that dietary intake of zinc is associated with the household income and educational status of caregivers/mothers. Similar to our finding in Ugandan children, consumption of meat and fish as well as their zinc intake was found high among communities of higher socioeconomic status.

One of the limitation in our study was, it didn’t consider calculating phytate zinc molar ratio for the dietary intake. The study did not estimate the contribution of breast milk to zinc intake among children still being breastfed and due to cost implication of dietary data collection, a single 24-h dietary recall was completed for each children. Therefore, intra-individual variation could not quantified.

## Conclusion

Dietary zinc intake among 6–35 months age children is very low in Ethiopia. Considering the high prevalence of stunting and roles of adequate zinc intake in supporting normal growth, nutrition and health planners need to emphasize on promoting zinc rich foods consumption and preventing childhood morbidity from common infections.

## Abbreviations

CI: Confidence Interval
EAR: Estimated Average Requirement
ENFCS: Ethiopian National Food Consumption Survey
Mg: Milligram
SD: Standard Deviation
SE: Standard Error
SNNPR: South Nations Nationalities and People Region
